# Human polyomaviruses identification by logic mining techniques

**DOI:** 10.1186/1743-422X-9-58

**Published:** 2012-03-02

**Authors:** Emanuel Weitschek, Alessandra Lo Presti, Guido Drovandi, Giovanni Felici, Massimo Ciccozzi, Marco Ciotti, Paola Bertolazzi

**Affiliations:** 1 Institute of Systems Analysis and Computer Science "A. Ruberti", National Research Council, Viale Manzoni 30, 00185 Rome, Italy; 2 Department of Informatics and Automation, Università degli studi Roma Tre, Via della Vasca Navale 79, 00146 Rome, Italy; 3 Department of Infectious, Parasitic and Immunomediated Diseases, Istituto Superiore di Sanità, Viale Regina Elena 299, 00161 Rome, Italy; 4 Laboratory of Molecular Virology, Foundation Polyclinic Tor Vergata, Viale Oxford 81, 00133 Rome, Italy

## Abstract

**Background:**

Differences in genomic sequences are crucial for the classification of viruses into different species. In this work, viral DNA sequences belonging to the human polyomaviruses BKPyV, JCPyV, KIPyV, WUPyV, and MCPyV are analyzed using a logic data mining method in order to identify the nucleotides which are able to distinguish the five different human polyomaviruses.

**Results:**

The approach presented in this work is successful as it discovers several logic rules that effectively characterize the different five studied polyomaviruses. The individuated logic rules are able to separate precisely one viral type from the other and to assign an unknown DNA sequence to one of the five analyzed polyomaviruses.

**Conclusions:**

The data mining analysis is performed by considering the complete sequences of the viruses and the sequences of the different gene regions separately, obtaining in both cases extremely high correct recognition rates.

## Background

Phylogenetic analysis is a technique used to perform species classification through DNA sequences analysis [1]. It examines sequence divergence or similarity through the alignment of DNA sequences. Recently, a new technique, named logic data mining, has been introduced and applied in species classification through DNA barcode, a short fragment of mitochondrial DNA composed of few hundreds of bases from which it is possible to extract the information needed to classify living species [2]. This technique allows finding patterns of nucleotides in the DNA barcode of a given species that characterizes it and allows distinguishing one species from the others. A pattern is defined as a set of positions of the DNA sequence whose corresponding nucleotides completely characterize the species. The logic data mining technique provides a sort of fingerprinting of the species and has been shown to allow a correct classification of new individuals. In this work we apply this technique to the classification of polyomaviruses. Human polyomaviruses are small double stranded DNA viruses of about 5 kb in length which belong to the *Polyomaviridae *family. Up to 2008, five human polyomaviruses have been identified and characterized: BK (BKPyV), JC (JCPyV), KI (KIPyV), WU (WUPyV) and MC (MCPyV) polyomaviruses. BKPyV and JCPyV were both uncovered in 1971. BKPyV was first identified in the urine of a kidney transplant patient [3], while JCPyV was uncovered in the brain tissue of a patient affected by progressive multifocal leucoencephalopathy (PML) [4]. The novel KIPyV and WUPyV have been identified in respiratory secretions of children with signs of acute respiratory disease [5,6], though there is little evidence that these viruses are the causative agents of respiratory disease. MCPyV was found integrated monoclonally in a rare skin cancer named Merckel cell carcinoma, strongly suggesting that viral infection may be an early event in the pathogenesis of Merkel cell carcinoma [7]. KIPyV, WUPyV and MCPyV share most of the genomic characteristics of BKPyV and JCPyV, with a noncoding control region (NCCR) separating the early and late coding regions on opposite strands [5-7]. However, unlike JCPyV and BKPyV, the three novel polyomaviruses lack of the gene region encoding for the agnoprotein.

The genome of each polyomavirus is about 5 Kb in length. It is important to adopt methods that are able to identify a subset of positions that characterize the polyomaviruses and the gene regions among the five analyzed polyomaviruses. The aim of this study is to identify nucleotide positions that allow distinguishing the five human polyomaviruses. The nucleotides, and their combinations, that determine an effective separation between the different polyomaviruses will be examined in a wider study for an effective biological analysis.

## Methods

### Sequences collection and alignments

All sequences of BKPyV, JCPyV, KIPyV, WUPyV and MCPyV deposited in Genbank until the date of submission of this paper were retrieved and analyzed by a logic data mining method (1982 total sequences). Of these, 40 sequences have been obtained in our laboratory and then deposited in GenBank. Before analysis, the sequences of each human polyomavirus type were aligned against the following reference sequences: BKPyV, NC_001538; JCPyV, NC_001699; KIPyV, NC_009238; WUPyV, NC_009539; MCPyV, NC_010277. Each gene fragment was aligned using Clustal × [8] and the sequences were manually edited with the Bioedit software [9]. Positions containing gaps were removed from the final alignment (each gene fragment was in frame and coded for the corresponding protein).

### The logic mining software system

The logic data mining software used in this study has been derived from previous works on the analysis of biological and genetic data [2,10-12] and it has been customized for the polyomavirus genome analysis. Its purpose is to identify logic rules, expressed as combination of the nucleotide positions, which are able to characterize univocally the different types of viruses in the same family. A standard classification paradigm is adopted, where the classification rules are extracted from a portion of the data (training set) and then tested on the remaining samples (test and verification data). Hereafter, we refer to the different types of viruses as classes to which the samples belong.

The input of the program is a FASTA format file of DNA sequences containing the training and testing sets. It is converted into an internal format named DMB that best organizes all the information needed for this analysis tool.

The main outputs of the software are:

• The logic rules that are able to individuate each type of virus, expressed as a combination of the nucleotide positions (e.g., the rule IF (pos437 = A) and (pos486 = C) THEN BK is to be interpreted as "if the nucleotide in position 437 is A and the nucleotide in position 486 is C then the sequence belongs to virus type BK").

• The classification statistics (confusion matrices, average and variances of error rates obtained with different sampling strategies).

The formulas are determined solving a series of optimization problems with the aim of minimizing their dimension and maximizing their precision.

**Figure 1:**
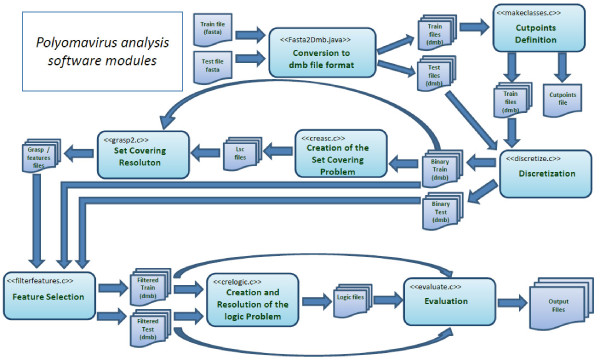
**Flow diagram of the logic mining software**.

The flow diagram of the logic mining software system in Figure 1 shows a schematic view of the architecture, representing the system flows and the fundamental modules. The main steps of the method are briefly described in the following paragraphs.

### The feature selection step

In this study the features are associated with a position that has a determinate nucleotide value (A, C, G, T). The aim of the feature selection step is to extract a small set of positions or species specific bases that are able to distinguish between the different species that are present in the data set. The selection of the most relevant features is a typical issue in data mining and data analysis [10,13]. We use an integer optimization model formulation to address the selection of the most relevant features. We consider a particular distance among samples and then select a given number of features that maximizes the smallest distances between each pair of samples of different classes. This optimization problem is of large size and grows quadratically with the number of sequences; for its solution a fast heuristic algorithm based on greedy randomized search is adopted. A detailed description of the method used to solve the underlying optimization problem can be found in references [2,10,11].

### The formula extraction step

The selected features in the previous step are taken as input to compute the logic separating formulas by the Lsquare method [12]. A logic formula is constituted by conjunctions ("and") and disjunctions ("or") of features. The classification formulas are extracted through the solution of an integer optimization problem based on a minimum cost satisfiability problem (MINSAT), which is solved by decomposition techniques. See reference [12] for further details. After obtaining the first set of logic formulas, the feature selection and the formula extraction steps are repeated to acquire all the separating formulas, adding a constraint that is able to avoid the selection of the previously chosen features. These two steps are iterated until no more separation is found.

### The application of the logic mining software

**Figure 2:**
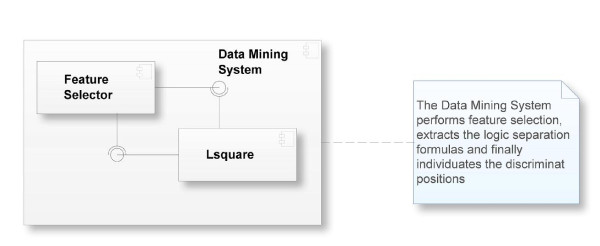
**Component diagram of the logic mining software**.

A typical application of the software is organized as follows. Each virus is defined as a *class*, indicated as *v*. For each *v*, a 2-class classification problem is defined, where class A contains the individuals of virus *v*, and class B the individuals of the other viruses. Then, the training data is analyzed in order to formulate the feature selection problem and to compute the optimal set of feature. Thereafter, the system identifies the logic formulas that separate the individuals in class A from those in class B. These formulas are then applied to the virus sequences, and, if a virus sequence is recognized as positive by the formula of species *v*, its predicted class is *v *and the prediction will be verified. When a virus is recognized by more than one formula (or by none of them) such an event is registered as a non classification. If a formula recognizes a virus of a class different from *v *into class *v*, we register such an event as a recognition error. Finally, the analysis is performed and a subset of patterns is produced. The component diagram in Figure 2 gives a final compact overview of the system.

## Results and discussion

### Logic mining analysis on each virus class

In order to understand the biological role of nucleotide sequences in a specific gene region of each polyomavirus, the analysis described in methods was carried out considering each virus as a class, in order to extract more general information. In this way, the focus is on positions that can discriminate between viruses. The number of sequences that compose the different gene regions and viruses is summarized in Table 1.

**Table 1:** Number of sequences

	LT	ST	VP1	VP2	VP3	TOTAL
**KIPyV**	8	27	10	8	8	61

**MCPyV**	13	28	3	2	2	48

**WUPyV**	14	16	14	23	14	81

**BKPyV**	0	0	192	192	192	576

**JCPyV**	0	0	406	405	405	1216

**TOTAL**	35	71	625	630	621	1982

The feature selection method was applied in order to select a small set of relevant positions. In the first runs a single position is selected only if it is able-just by itself-to distinguish between the groups. After a position is chosen and the logic formulas are obtained, the selected position is removed from the data, reiterating this procedure until no separation with only one feature is possible. A 100 fold cross validation sampling of the different sequences was performed to validate the results. The original sequences were randomly partitioned into 100 folds and the validation was performed 100 times, each on different sequences.

The adoption of this approach results in the discovery of the most characteristic base pairs of the data set. The method led to the identification of formulas each with the property of being able to correctly classify a polyomavirus.

In this step the main purpose of the analysis was to distinguish the five different polyomaviruses (BK, JC, KI, MC, WU) in all the 1982 sequences (here all the genes are considered together). The classification has a perfect recognition rate (100% in training and in test set). The logic formulas are listed in Table 2, where we note that they are composed by few literals (from three to five) and all formulas have only one clause in conjunctive normal form (i.e., a sequence of conjunctions).

**Table 2:** Logic formulas for virus classification.

Species	Genes	Formulas	Coverage
BK	VP1,VP2,VP3	(pos437 = A) AND (pos486 = C)	1.00

JCV	VP1,VP2,VP3	not(pos338 = C) AND (pos532 = C)	1.00

KIV	ST, LT,VP1,VP2,VP3	not(pos294 = T) AND not(pos358 = T) AND not(pos521 = T) AND not(pos532 = G)	1.00

MCV	ST,LT	(pos199 = A) AND not(pos286 = T)	1.00

WUV	ST, LT, VP1, VP2,	not(pos286 = T) AND pos425 = A AND not(pos474 = G)	1.00

The different nucleotide positions are calculated from the ATG starting codon of each gene and are shown in parentheses

We recall the straight-forward interpretation of the formulas of Table 2: in the fourth row of the Table, we read that "if position 199 is A, and if position 286 is different from T, it is a MC polyomavirus". The last column of Table 2 reports a measure of the coverage of the formula; it indicates the proportion of samples of that class that are correctly classified. When it is equal to 1, as it is the case for all formulas but two, it means that that single formula performs exact classification of the training data.

### Logic mining analysis over 21 gene regions

Another classification analysis was done by distinguishing the different 21 gene regions and polyomaviruses in all the 1982 sequences. The available gene regions were Small t antigen (ST), Large t antigen (LT), VP1, VP2 and VP3. For performing the analysis and to validate the results a 100 fold cross validation sampling of the different sequences was applied. Also in this analysis the recognition rate is very high (99% both in training and testing set). The logic separating formulas are listed in Table 3. We point out that the error rate of 1% is caused by the very small amount of sequences available in MCPyV-VP1, MCPyV-VP2 and MCPyV-VP3: 3, 2 and 2 respectively. In this case the logic formulas are very compact (from one to three literals) and precise.

**Table 3:** Logic formulas for gene-virus classification

Species	Formulas	Coverage
BKVP1	(pos504 = T) AND (pos518 = C)	1.00

BKVP2	(pos410 = T) AND (pos554 = A)	1.00

BKVP3	(pos518 = A) AND (pos521 = G)	1.00

JCVP1	(pos410 = G) AND (pos466 = T)	1.00

JCVP2	(pos383 = A) AND (pos417 = G)	1.00

JCVP3	(pos161 = G) AND (pos406 = A)	1.00

KIVLT	(pos417 = G) AND (pos472 = C)	1.00

KIVST	(pos360 = T) AND (pos381 = A)	1.00

KIVP1	(pos239 = C) AND (pos457 = G)	1.00

KIVP2	(pos518 = C) AND (pos547 = A)	1.00

KIVP3	(pos406 = G) AND (pos472 = C)	1.00

MCVLT	(pos457 = C) AND (pos547 = C)	1.00

MCVST	(pos417 = A) AND (pos504 = T)	1.00

MCVP1	(pos521 = C) AND (pos547 = A)	1.00

MCVP2	(pos521 = C) AND (pos547 = A)	0.00

MCVP3	(pos521 = C) AND (pos547 = A)	0.00

WUVLT	(pos547 = A) AND (pos554 = A)	1.00

WUVST	(pos504 = G) AND (pos554 = A)	1.00

WUVP1	(pos122 = C)	1.00

WUVP2	(pos521 = C) AND (pos554 = A)	1.00

WUVP3	(pos518 = C) AND (pos547 = G)	1.00

### Logic mining analysis in the same gene region

We also performed the virus classification within the same gene regions, adopting a 100 fold cross validation approach, and found the separating formulas between the different viruses in that particular gene region. The viruses differ in multiple positions and we found all the discriminating base pairs. We remand to the appendix [Additional file 1] for further explanation of the experiments and to Additional file 2 and Additional file 3 for visualizing the logic formulas.

## Conclusions

In this work the human polyomaviruses genome was analyzed using a logic data mining approach in the attempt to get insights into the specific features of the viral sequence. The objective of the analysis was to identify very small portions of the DNA sequence, composed by one or few nucleotides, which are able to characterize the different viral types. The approach appears to be successful as it identifies several small "rules" that effectively characterize the different classes of polyomaviruses and that are able to separate one class from the other with utter precision. The analysis was carried out on both the virus sequences as a whole and the different gene regions, obtaining in both cases extremely high correct recognition rates. In particular, for each polyomavirus (BK, JC, KI, MC, WU), a single logic formula was able to provide a perfect classification; in addition, within the same gene region we discovered the logic formulas that can distinguish all the different polyomaviruses with a 99% accuracy.

The first classification experiment was based on all the gene regions of all five different polyomaviruses sequences aligned from the ATG starting codon. This experiment led to the identification of some particular positions of a polyomavirus for all the gene regions; these positions enable to distinguish that polyomavirus from the others. In other words, all the gene regions of a given polyomavirus exhibit a prevalence of an allele with respect to the same positions in all the gene regions of the other viruses. This evidence directs further studies towards the understanding of the biological meaning of those particular positions.

Recently, taxonomical developments in the family *Polyomaviridae *have been proposed. According to these suggestions the family *Polyomaviridae*, which is currently constituted as a single genus polyomavirus, will include three genera: two containing mammalian viruses and one containing avian viruses. The two mammalian genera are named Orthopolyomavirus and Wukipolyomavirus, while the avian genus is named Avipolyomavirus.

The demarcation criterion used for the new proposed polyomavirus species is based on the evaluation of the whole-genome nucleotide sequence identity respect to members of known species with less than 81% similarity [1]. With the method proposed in this work, it is possible to distinguish the five described human polyomaviruses focusing only on few nucleotide positions. This approach may thus help defining new viral species (i.e., when interpreting sequences obtained by high-throughput sequencing) in conjunction with the classical method based on sequence identity.

Overall, this is a promising approach that needs to be validated on a larger sample size. For instance, it will be interesting to verify how mutations (polymorphisms, deletions and insertions) in different genomic regions can affect this analysis. To this regard, we are going to select and analyze sequences obtained from patients with known mutations in particular regions or domains of the viral genome [14].

## Abbreviations

A: Adenine; BK: B.K. (patient initials); BKPyV: B.K. (patient initials) polyomavirus; C: Cytosine; DNA: Deoxyribonucleic acid; G: Guanine; JC: John Cunningham; JCPyV: John Cunningham polyomavirus; KI: Karolinska Institute; KIPyV: Karolinska Institute polyomavirus; LT: Large t antigen; MC: Merkel Cell; MCPyV: Merkel Cell polyomavirus; NCCR: Noncoding control region; PML: Progressive multifocal leucoencephalopathy; ST: Small t antigen; T: Thymine; VP1: Gene region VP1; VP2: Gene region VP2; VP3: Gene region VP3; WU: Washington University; WUPyV: Washington University polyomavirus.

## Competing interests

The authors declare that they have no competing interests.

## Authors' contributions

PB, MCC, MCT and GF designed research. ALP performed sequences retrieval and alignments. EW, GD and GF engineered the software. EW, GF and ALP planned and revised the statistical analysis. EW and ALP performed the analysis. All authors contributed equally in writing the paper. All authors read and approved the final manuscript.

## Supplementary Material

Additional file 1 **Appendix**. Test Plan and statistical experiments. Click here for file

Additional file 2 **Separating formulas for LT gene region**. All the discriminating base pairs for the virus classification within the gene region LT. Click here for file

Additional file 3 **Separating formulas for ST gene region**. All the discriminating base pairs for the virus classification within the gene region ST. Click here for file
