# Supplementary material for: Human polyomaviruses identification by logic mining techniques
Source: Virol J. 2012 Mar 2;9:58. doi: 10.1186/1743-422X-9-58 (PMC3307486; doi:10.1186/1743-422X-9-58)
Supplement: Additional file 1 — Appendix. Test Plan and statistical experiments. [file 1743-422X-9-58-S1.PDF]

Annex to Human polyomaviruses genome analysis by logic mining techniques

Test Plan

The experimentations have been performed with the logic data mining software for DNA sequences analysis, designed and developed by the authors.
For extracting the logic separating formulas the sequences have been divided in disjoints sets, training set and testing set, using a 100-fold cross validation. The test set is used for sounding the logic classification formulas.

We performed the following experiments:

I. Virus classification

The goal of this test is to distinguish the different viruses by considering:

a) All genes together

b) Single genes

II. Virus and gene classification

In this test the goal is to distinguish the gene – virus pairs (KIVST, KIVLT, MCVST…) by considering:

a) All the gene – virus pairs

Number of sequences

In the following tables we can see the number of sequences of the data set

	LT	ST	VP1	VP2	VP3	TOTAL
**KIV**	8	27	10	8	8	61
**MCV**	13	28	3	2	2	48
**WUV**	14	16	14	23	14	81
**BK**	0	0	192	192	192	576
**JCV**	0	0	406	405	405	1216
**TOTAL**	35	71	625	630	621	1982

Polyoma-viruses and gene regions

I. Virus classification

a) All genes together (virus classification)

The aim of the classification is the distinction of the five types of polyoma viruses (KIV, MCV, WUV, BK, JCV) in all 1982 sequences (the genes are considered all together)

The classification performance is the following:

100 % of correct classified elements in training set

100 % of correct classified elements in test set

The logic formulas are the following:

Species	Genes	Formulas	Coverage
BK	VP1,VP2,VP3	(pos437=A) AND (pos486=C)	1.00
JCV	VP1,VP2,VP3	not(pos338=C) AND (pos532=C)	1.00
KIV	ST, LT,VP1,VP2,VP3	not(pos294=T) AND not(pos358=T) AND not(pos521=T) AND not(pos532=G)	
MCV	ST,LT	(pos199=A) AND not(pos286=T)	1.00
WUV	ST, LT, VP1, VP2,	not(pos286=T) AND pos425=A AND not(pos474=G)	1.00

In the virus classification process, where we consider all the different genes together, we are able to separate completely the different polyoma viruses with a single separating logic formula for every class of virus (BK, JCV, KIV, MCV, WUV).

b) Single genes (virus classification in the same gene region)

The aim of the classification is the distinction of the five types of polyoma viruses (KIV, MCV, WUV, BK, JCV) by considering the different gene regions separately

LT gene region (35 sequences, 3 different polyoma viruses)

The classification performance is the following:

100 % of correct classified elements in training set

100 % of correct classified elements in test set

The logic formulas for every class are listed in the file LT.xls

ST gene region (71 sequences, 3 different polyoma viruses)

The classification performance is the following:

100 % of correct classified elements in training set

100 % of correct classified elements in test set

The logic formulas for every class are listed in the file ST.xls

VP1 gene region (625 sequences, 5 different polyoma viruses)

The classification performance is the following:

100 % of correct classified elements in training set

100 % of correct classified elements in test set

The logic formulas of the different classes are the following:

Species	Formulas	Coverage
BKVP1	(pos1054=G)	1.00
JCVVP1	(pos285=G)	1.00
KIVVP1	(pos53=A)	1.00
MCVVP1	(pos410=C)	1.00
WUVVP1	(pos1054=T)	1.00

VP2 gene region (630 sequences, 5 different polyoma viruses)

The classification performance is the following:

100 % of correct classified elements in training set

100 % of correct classified elements in test set

The logic formulas of the different classes are the following:

Species	Formulas	Coverage
BKVP2	(pos109=G)	1.00
JCVVP2	(pos723=T)	1.00
KIVVP2	(pos96=T)	1.00
MCVVP2	(pos639=G)	1.00
WUVVP2	(pos683=T)	1.00

VP3 gene region (621 sequences, 5 different polyoma viruses)

The classification performance is the following:

100 % of correct classified elements in training set

100 % of correct classified elements in test set

The logic formulas of the different classes are the following:

Species	Formulas	Coverage
BKVP3	(pos590=C)	1.00
JCVVP3	(pos590=G)	1.00
KIVVP3	(pos284=C)	1.00
MCVVP3	(pos13=G)	1.00
WUVVP3	(pos590=T)	1.00

In the virus classification process for sequences of the same gene regions we can find all the single separating positions for the gene regions LT and ST. For the other gene regions (VP1, VP2 and VP3) we need more positions for separating the different polyomaviruses.

II. Virus and gene classification

a) All the gene – virus pairs

The aim of the classification is the distinction of the 21 types of gene regions and polyoma viruses in all 1982 sequences

The classification performance is the following:

99 % of correct classified elements in training set

99 % of correct classified elements in test set

The logic formulas of the different classes are the following:

Species	Formulas	Coverage
BKVP1	(pos504=T) AND (pos518=C)	1.00
BKVP2	(pos410=T) AND (pos554=A)	1.00
BKVP3	(pos518=A) AND (pos521=G)	1.00
JCVP1	(pos410=G) AND (pos466=T)	1.00
JCVP2	(pos383=A) AND (pos417=G)	1.00
JCVP3	(pos161=G) AND (pos406=A)	1.00
KILT	(pos417=G) AND (pos472=C)	1.00
KIST	(pos360=T) AND (pos381=A)	1.00
KIVP1	(pos239=C) AND (pos457=G)	1.00
KIVP2	(pos518=C) AND (pos547=A)	1.00
KIVP3	(pos406=G) AND (pos472=C)	1.00
MCLT	(pos457=C) AND (pos547=C)	1.00
MCST	(pos417=A) AND (pos504=T)	1.00
MCVP1	(pos521=C) AND (pos547=A)	1.00
MCVP2	(pos521=C) AND (pos547=A)	0.00
MCVP3	(pos521=C) AND (pos547=A)	0.00
WULT	(pos547=A) AND (pos554=A)	1.00
WUST	(pos504=G) AND (pos554=A)	1.00
WUVP1	(pos122=C)	1.00
WUVP2	(pos521=C) AND (pos554=A)	1.00
WUVP3	(pos518=C) AND (pos547=G)	1.00

In the gene – virus classification process we can separate the different classes with an accuracy of 99 % by considering all the gene – virus pairs.
